# Analysis of Predictive Factors on Minors’ Mental Health According to the Spanish National Health Survey

**DOI:** 10.3390/brainsci7100135

**Published:** 2017-10-21

**Authors:** Fernando Fajardo-Bullón, Irina Rasskin-Gutman, Elena Felipe-Castaño, Eduardo João Ribeiro dos Santos, Benito León-del Barco

**Affiliations:** 1 Department of Psychology, Faculty of Education and Psychology, University of Extremadura, Badajoz 06006, Spain; 2 Department of Psychology, Faculty of Teacher Training College, University of Extremadura, Cáceres 10071, Spain; irasskin@unex.es (I.R.-G.); efelipe@unex.es (E.F.-C.); bleon@unex.es (B.L.-d.B.); 3 Scientific Coordinator R&D Unit Institute of Cognitive Psychology (IPCDHS/FPCE), University of Coimbra, Coimbra 3000-115, Portugal; eduardosantos@fpce.uc.pt

**Keywords:** mental health, child welfare, social class, health promotion

## Abstract

Research on minors’ mental health is an increasingly developing area. Given the increased prevalence of disorders, it seems necessary to analyze the factors that can affect poor mental health. This study analyzes the influence of occupational class, educational level, age, sex and perceived mental health of Spanish children, which is measured through the Strengths and Difficulties Questionnaire. The sample consists of 3599 minors between 4 and 14 years old, who were interviewed through the Spanish National Health Survey 2011. Our results indicating the significant (*p* < 0.05) relationship between mental health, occupational class (OR 0.533) and minors’ health in the last year (OR 0.313) are shown. However, gender (OR 1.187) and educational level of Pre-School Education in relation to Secondary Education (OR 1.174) and Primary Education (OR 0.996) do not generate significant differences. In conclusion, we consider it necessary to design and implement public policies aimed at improving the care system for children who have had poor or regular health in the last year, and whose parents are positioned in the lowest part of the occupational scale.

## 1. Introduction

For more than a decade, the problem of mental health in children, with rates of those affected being between 10% and 20%, worldwide, has increased [1,2]. We know that new generations are now more likely to have mental health problems than previous ones, and that inadequate mental health may be linked with suicide cases, which are the second and third causes of teenage mortality, according to several international studies reviewed [3]. If, at the same time, we take into account that 50% of adults’ disorders had their onset in adolescence, it seems necessary to perform an analysis of the factors that affect the mental health of minors, not only as a treatment measure, but also with the aim of preventing future disorders [4,5]. In fact, the presence of mental health problems in the parents could be a predictor of mental health problems in children, and therefore this situation would form a cycle where the difficulties could pass from parents to children [6].

Two main difficulties for this analysis are the use of different questionnaires that make difficult to compare the international samples studied, as well as the fact that local studies do not allow the generalization of the results. To overcome these obstacles, the Strengths and Difficulties Questionnaire (SDQ)—parents version [5,7]—has been implemented in this study. This questionnaire has been used with a Spanish population previously [6,8,9], and has demonstrated good qualities for measuring mental health at a national and international level, especially with its scoring at the total scale in difficulties [7,10]. In the present study, we use the same scale as was included in the Spanish National Health Survey (ENSE, 2011–2012) [8,11]. This questionnaire, in its ‘parents’ version, has proved to be a good tool for the screening of mental disorders in cross-cultural studies in European countries, establishing itself as an international mental health measurement tool [12].

Several reports indicate the need to analyze which variables affect the mental health of minors, and encourage the social sciences to collect data in order to clarify the increase of mental health problems in minors in Western countries [2,10,13,14,15]. According to previous analyses with Spanish national samples, it seems that the occupational class of parents could be a relevant variable for the mental health of minors [16], with less favored classes predicting worse results in the mental health of minors. As in North America, those with low-income parents associated with low occupational classes had higher levels of depression and anxiety [17], and may even experience feelings of helplessness and inferiority, or behaviors of alcohol abuse [18]. Given the economic crisis in Spain since 2008, it seems necessary to analyze the variables that may affect the mental health of Spanish minors. Therefore, the objective of this study is to analyze the influence of variables such as “occupational class of the parents”, “educational level”, “sex” and “perceived health”—according to the 2011 Spanish National Health Survey—on the mental health of Spanish children aged between 4 and 14 years old.

## 2. Materials and Methods

### 2.1. Participants

The Spanish National Health Survey (ENSE-2011/12) [11] collects health information relative to the resident population in Spain in 21,508 households, these being the main dwellings. The sample size was 26,502 interviews, 21,007 adults (15 and over), and 5495 children (0–14 years), the latter by interviewing their parents/guardians. In this study, we have worked exclusively with the results of the survey related to minors from 4 to 14, and whose responses were obtained by their parents. Therefore, our study is based on a sample of 3599 children. 52.49% male, 17.45% between 4 and 5 years old, 62.24% between 6 and 12 years old, and 20.31% between 13 and 14 years old (Table 1). These data were collected between July 2011 and June 2012. In order to achieve the goals of the survey and provide estimation with a certain degree of reliability (both in national and regional levels), a sample of 24,000 dwellings distributed in 2000 census sections was selected. There were 12 dwellings selected in each census section. To determine the size of the sample, the type of characteristics studied, the information provided by the selected respondents, and the importance of the representativeness of the study of children were taken into account. The sample was distributed among the regions (Spanish Autonomous Communities), assigning one equitable part and another taking into account the proportion of the region size. Sections were selected for each stratum based on the probability proportional to their size. That is to say that the dwellings in each section would have the same probability via random systematic sampling, and the procedure allowed self-weighted samples in each stratum to be obtained. More information regarding this procedure is available from the Ministry of Health, Social Services and Equality.

### 2.2. Instrument

The instrument used was the SDQ-parent questionnaire [7]. It has been translated into 66 languages and internationally validated [9,19,20]. It is an instrument of excellent quality for the screening of mental health in minors, and its usability, as well as the reliability of the scores, makes it very attractive for research [21,22]. It consists of 5 scales of 5 items each. The total score for difficulties is calculated based on the sum of the first 4 scales (emotional symptoms, behavior problems, hyperactivity, problems with peers), avoiding the sum of the last scale (prosocial), as has been established in the methodology of previous research using the SDQ, including that of Goodman and Goodman, its creators [22]. To facilitate the analysis of mental health, once the answers had been obtained, the score of each minor—that would vary between 0 and 40 points—was divided into two categories: suffering or not suffering from mental health problems, based on whether the total score was greater than or equal to 20 points (suffer) or less than 20 points (not suffering) [9,23]. This variable was contrasted with the complementary sociodemographic information obtained through interview: occupational class, educational level, sex, and perceived health. Six occupational social classes were considered, based on the Spanish adaptation of the British Registrar General classification [24]. The authors grouped them into three social classes for better study: class 1 (combining the most privileged social classes I and II), class 2 (combining the middle classes III and IVa) and class 3 (combining classes less privileged in that register; IVb and V). Additionally, based on the minors’ age, three corresponding educational levels were considered: Pre-School Education (children between four and five years old), Primary Education (minors between six and 12 years old) and Compulsory Secondary Education (ESO) (13 and 14 years old). For the perceived health in the last year, three levels were considered: “good or very good”, “regular”, and “bad or very bad”.

### 2.3. Procedure

The type of sampling used was stratified tri-stage. The information corresponding to the questionnaire for minors was obtained indirectly, facilitated in our case by the mother or the father. The method of collecting information was Computer-Assisted Personal Interviews (CAPI), direct in the case of adults, and the mother/father or guardian. This is to say that, in our study, we have only selected those responses by parents of the minors. This survey was approved by the Committee of Good Practices of the European Statistical System (ETUCE) under the protocol of action of the National Institute of Statistics and Ministry of Health Social Services and Equality developed by ENSE-2011 (Directive 95/46/Parliament and the European Council of 24 October 1995 on the protection of individuals with regard to the processing of personal data and on the free movement of such data).

### 2.4. Analyitic Approach

Analysis of variance techniques (ANOVA) were used to analyze whether there were significant differences between the total score obtained in the SDQ-parent test and the independent variables or selected factors, such as sex, educational level, occupational class, and perceived health. In order to analyze the association between having or mental health problems or not with respect to the risk factors mentioned above, we used the statistical tools of odds ratio (OR) and relative risks (risk ratio, RR). In this last analysis, we used the occupational social class 3 (less privileged) and Compulsory Secondary Education as comparative control classes. For the regression analysis, 189 subjects with missing or incomplete observations were eliminated. The remaining subjects in the sample were all included. 

## 3. Results

In Table 1, the frequency distribution and percentages of the mean of the scores reached in the SDQ test, ‘parent version’, can be observed, according to the risk factors chosen.

A priori, there appear to be significant differences in means in the sex factor, the educational level factor, the occupational class factor and among the three categories of the perceived health factor (Table 2).

Through the analysis of a general univariate linear model, the sex factor (*F* = 14.372; *p* = 0.000) and the educational level factor show significant differences (*F* = 5.388, *p* = 0.0048). Scheffé’s post hoc tests show that these significant differences exist between Pre-School Education and all other categories (Primary and Secondary Education), and there are no significant differences between the categories Primary Education and Secondary Education. In addition to this, minors who are in Pre-School Education have a higher propensity to suffer mental health problems.

As far as the occupational class is concerned, there were significant differences between the means of their categories (*F* = 26.391, *p* = 0.000) and there were significant differences among the three occupational level classes in Scheffé’s post hoc tests.

Finally, in relation to perceived health in the last year, significant differences were obtained between the means of perceived health levels (*F* = 51.172, *p* = 0.000). Furthermore, through Scheffé’s post hoc tests, different means were obtained in the three levels, with minors who presented poor or very bad perceived health during the last year being the most likely to have mental health problems.

The results given in Table 2 present the measurements of the linear relationships between the total score obtained in the test and the risk factors (sex, age, occupational class and perceived health).

Risk factors explain 5.5% of the variability of the total score of the SDQ, but the influence of all of them is very significant, together (test *F* = 40.007) and separately (*t*-statistic).

On the other hand, taking into account the risk factors arises the interest in calculating the odds ratios and relative risks of being ill or healthy (Table 3).

## 4. Discussion and Conclusions

According to the results obtained in this study, it seems that, although the sex, the age associated with the corresponding educational level, the perception of health in the last year, and the occupational class are factors that affect the total score in mental health difficulties (SDQ-Parents), it is exclusively the health over the last year and the occupational class that have the capacity to influence the distinction, at a diagnostic or screening level, between sick and healthy.

Regarding sex, boys are more likely to have higher overall mental health scores than younger women. However, these differences disappear when we use the cut-off points of healthy and sick for the Spanish population. They may have worse symptomatology, but at the time of either diagnosis or more thorough screening, it seems that there are no significant differences, as this agrees with the data obtained in the ENSE-2006 [9,25]. Although it is not the goal of this paper, other studies have shown that, when analyzing pathologies not globally, but in terms of scale, there is a greater symptomatology of behavioral disorders and externalizing symptoms in boys at an early age, and a more frequent occurrence of symptoms of eating disorders, depression and internalizing symptoms in girls as they progress toward adolescence [13,26,27].

On the other hand, if the child’s educational level is taken into account, minors in the Pre-School stage are at higher risk of having mental health problems than those in primary education or secondary education, with primary education having a higher risk than Secondary Education. These results are in agreement with other studies that involved measurements of total SDQ scores of children between 6 and 18 years old, and where the highest scores were in the lowest ages [16]. Even so, the scientific literature is quite divergent with respect to the effects of age on the mental health of the minors or, in this case, of age associated with academic level [26]. However, when we analyzed the ability of this factor to discriminate between healthy or sick, which had not been carried out by previous investigations in Spain with this sample, no differences based on the educational level of the child could be observed. These results are in agreement with those obtained in the Spanish National Health Survey 2006, previous to the one analyzed here [28]. At the same time, factors such as “low parental education” may be a factor that negatively affects children’s mental health, especially in the early stages (4–11), without affecting later stages at adolescence [29].

Some authors [30,31] point to socioeconomic status and parental education [32] as the most common causes of mental health problems in minors. This study shows the existence of an association between parents’ lower occupational class and an increased probability of suffering mental health problems in minors. The most qualified occupational class offers a factor of protection in comparison to the unqualified working class, which is in agreement, again, with the data of the ENSE-2006 [16]. Other studies confirm the influence of parents’ low occupational class on the mental health of the children in the Spanish and international population [29,33], even specifying how behavioral disorders or problems of hyperactivity, emotional problems, and peer problems increase in children that belong to these families [28,34]. It seems that occupational class and, even more, the presence of unemployment, both associated with low income, may be factors that favor the appearance of mental health problems in Spanish minors [28], having the capacity to to enhance depression in adolescent girls and alcohol abuse behaviors in boys [18,35]. At the same time, there is a positive relationship between the occupational class and quality of life, with this factor having an impact on the promotion of health problems, not only mental, but also physical [34]. In this sense, the significant differences of this study show how poor health in the last year favors the possibility of suffering mental health problems. Health perceived as good or very good is a protective factor against the occurrence of mental health problems, and this remained the same in both health surveys, 2006 and 2011 [9]. These results also coincide with other recent research that advocates the association between good mental health and good physical health [4]. This could indicate the need to analyze mental health when the child complains of a poor physical health in the last year, which would imply the relevance of primary care in health centers for the detection of mental health problems. At the same time, it seems that a poor socioeconomic situation, associated with a low occupational class, may influence not only poor mental health, but also poor physical health and lifestyle [15,36]. It therefore appears that socioeconomic status, associated with the occupational class of the parents, can influence mental health and, in turn, the physical health of minors [36,37]. Although differences in mental health problems have not been perceived in minors before and after the Spanish economic crisis [38], it does seem important to highlight how occupational class remains a determinant factor for their mental health in both periods. In fact, a low socioeconomic status is related to major mental health problems in parents and, as a result, to mental health problems in minors [9,30].

It is important to emphasize that, despite the strength of this study in carrying out a national population analysis under a rigorous methodology, it would be advisable to resolve its limitations through the use of the SDQ questionnaire together with clinical interviews offering information complementary to that obtained from the use of the questionnaire.

Thanks to the results of this study, it can be concluded that the low occupational class of the parents [39], connected with the poor health of the child in the last year [25], may be risk factors for Mental Health Problems in minors. 

Although there was a decrease in the psychological and emotional problems of Spanish children between 2006 and 2012 [1,2], it is noteworthy that these factors remained relevant to the Spanish population, whether before (ENSE-2006) or during the economic crisis (ENSE-2011/12). Therefore, social policies are needed for detecting those families with these variables in order to be able to work with this population and to prevent future pathologies in mental health [2].

## Figures and Tables

**Table 1:** Descriptive statistics according to the studied variables.

Variables	N (%)	^a^ SDQ’s Ratings M (DT)
Sex		
Male	1889 (52.49)	8.78 (5.53)
Female	1710 (47.51)	8.09 (5.43)
Total	3599 (100)	8.45 (5.49)
Education		
Pre-School (4–5 years old)	628 (17.45)	9.08 (5.33)
Primary (6–12 years old)	2240 (62.24)	8.34 (5.49)
Secondary (13–14 years old)	731 (20.31)	8.24 (5.61)
Total	3599 (100)	8.45 (5.49)
Occupational Class		
Class 1	720 (21.11)	7.38 (4.95)
Class 2	1172 (34.37)	8.27 (5.41)
Class 3	1518 (44.52)	9.13 (5.66)
Total	3410 (100)	8.46 (5.47)
Perceived Health		
Good or very good	3367 (93.58)	8.22 (5.31)
Regular	211 (5.86)	11.60 (6.72)
Bad or very bad	20 (0.56)	14.50 (7.30)
Total	3598 (100)	8.45 (5.49)

^a^ SDQ = Strengths and Difficulties Questionnaire.

**Table 2:** Ordinary Least Squares Model.

	Coefficient	DT	*t*	*p*
Constant	4.8829	0.5903	8.2705	<0.0001
Sex	−0.7253	0.1826	−3.9714	<0.0001
Occupational Class	0.8356	0.1177	7.0974	<0.0001
Perceived Health	3.3601	0.3283	10.2342	<0.0001
Age	−0.2728	0.0684	−3.9863	<0.0001

*R*^2^ = 0.055 (5.5%); *F*(3,3404) = 40.007; Value *p* < 0.001.

**Table 3:** Ratios and Odds Ratios depending on the different risk factors.

Risk Factor	Rate ^a^	Observed		IC (95%)	χ2	*p*
		RR ^b^	OR ^c^			
Sex						
Male Female	0.094 0.080	1.169	1.187	(0.945–1.447) (0.940–1.499)	2.08	0.14
Educational Level						
Pre-School Secondary	0.094 0.081	1.158	1.174	(0.874–1.530) (0.862–1.595)	1.03	0.31
Educational Level						
Pre-School Secondary	0.093 0.094	0.997	0.997	(0.679–1.303) (0.651–1.341)	0.13	0.72
Occupational Class						
Executive Skilled	0.058 0.083	0.705	0.686	(0.497–1.001) (0.472–0.998)	3.91	0.99
Occupational Class						
Executive Non Skilled	0.058 0.104	0.560	0.533	(0.403–0.778) (0.375–0.759)	12.56	<0.05
Perceived Health						
Very well/well Regular	0.078 0.213	0.366	0.313	(0.276–0.486) (0.220–0.445)	46.11	<0.05
Perceived Health						
Very well/well Very bad/bad	0.078 0.300	0.260	0.198	(0.132–0.514) (0.075–0.519)	*	

^a^ Rate = Proportion in the risk factor group with presence of mental health problems; ^b^ Ratio Risk (RR) = Rate (1)/Rate (2); Odds (1) = present (1)/absent (1), Odds (2) = present (2)/absent (2); ^c^ Odds Ratio (OR) = Odds (1)/Odds (2). ^*^ Frequencies less than five or empty.
